# Testicular and Color Variation in the Kissing Bug, *Rhodnius brethesi*, in Amazonas, Brazil

**DOI:** 10.1673/031.012.6501

**Published:** 2012-05-21

**Authors:** Simone P.C. Freitas, Sandra F. Bonifácio, Ângela C.V. Junqueira, Ana L.B. Souza, Teresa C.M. Gonçalves

**Affiliations:** ^1^ Setor de Entomologia Médica e Forense, lnstituto Oswaldo Cruz, FIOCRUZ, Rio de Janeiro, Brazil, 21045900; ^2^ Laboratório Central, Universidade Federal de São Paulo, UNIFESP, São Paulo, Brazil, 04024002; ^3^ Laboratório de Doenças Parasitárias, lnstituto Oswaldo Cruz, FIOCRUZ, Rio de Janeiro, Brazil, 21045900; ^4^ Departamento de Ciências Biológicas, Universidade Estadual do Sudoeste da Bahia, Jequié, Bahia, Brazil, 45206510

**Keywords:** male reproductive system, morphology, testis, vector

## Abstract

Because of the morphological and morphometric variation of testicular follicles in different genera of the subfamily Triatominae, it was of interest to associate those parameters with the different medial pronotal band patterns (wide and narrow) found in *Rhodnius brethesi* (Matta) (Hemiptera: Reduviidae). This is a wild species often associated with *Leopoldina piassaba* Wallace (Arecales: Arecaceae) palm, with a geographical distribution restricted to the Amazon region. The specimens used were from the state of Amazonas, and were kept under conditions of 29 ± 1 ^°^C, 80 ± 5% RH, 12:12 L:D photoperiod, and were fed weekly on blood from Swiss mice. Three—day—old fasting males were separated in accordance with the patterns of the medial pronotal band, dissected, and the testicles removed. After removal of the testicular membrane, the follicles were spread, drawn by camera lucida, and measured. The results showed that the testis of *R*. *brethesi* consists of seven follicles, divided into two groups by length; two long and five short. In specimens with a wide medial pronotal band, the long follicles were 5.4 mm in length, but in specimens with a narrow medial band, the long follicles were 5.64 mm in length. The difference was significant. The short follicles were not different in length, suggesting the presence of a possible complex “brethesi” in the Amazon region.

## Introduction

The male reproductive system in Triatominae has two testes connected to the aedeagus by a pair of vas deferens. Each testis is an oval structure lined by a scrotal membrane that encloses seven tubules known as the testis follicles. Each follicle is narrowed in the proximal region forming a short canal that converges to the vas deferens. In the medium region, the vas deferens dilates to form the seminal vesicle, and then acquires its normal width until it approaches the ejaculatory duct, which terminates in the aedeagus (Freitas et al. 2008). Associated with this system are the four pairs of accessory glands of mesodermal origin (Freitas et al. 2010).

Testicular size shows individual variations among specimens, and according to age, feeding condition and reproductive activity (Barth 1956). Similarly, follicles have different lengths; thus, morphometric dimensions of these structures enable the differentiation among genera (Gonçalves et al. 1987), and have even offered morphometric validity supporting the taxonomical return of the species *Triatoma spinolai* to genus *Mepraia* (Lent et al. 1994).

Schreiber et al. (1968), Silva and Schreiber (1971), and Gonçalves et al. (1987), classified genera into categories according to the number, size, and thickness of the testis follicles: *Panstrongylus* has seven narrow follicles of similar lengths, *Rhodnius* and *Psammolestes* have five short and fine follicles as well as two medium and thick follicles, and *Triatoma* has three short and narrow, two medium and thick, and two long and narrow follicles. *Triatoma rubrofasciata* is the exception within the genus, with four categories of follicles: one long, two medium,two short, and two very short (Freitas et al. 2007), showing interspecific differences of *within*—*Triatoma* pattern.

Based on these data, Freitas et al. (2008) analyzed the morphometry of the testis follicles of four color patterns of the *Triatoma brasiliensis* complex, classified into three species: *T*. *brasiliensis*, *T*. *melanica*, *T*. *juazeirensis*, and one subspecies *T*. *brasiliensis macromelasoma* (Costa et al. 2003; Costa et al. 2006; Costa and Félix 2007). The results showed three categories of follicles: three short, two medium, and two long, as described for the typical *Triatoma*. However, according to statistical analyses, *T*. *brasiliensis* and *T*. *melanica* showed significant differences for each category of the follicles, while *T*. *juazeirensis* and *T*. *br*. *macromelasoma* had similar lengths within each category.

The present study examined testicular follicles of *Rhodnius brethesi* (Matta) (Hemiptera: Reduviidae), which is found in association with *Leopoldina piassaba* Wallace (Arecales: Arecaceae) palm tree (Matta 1919). Coura et al. (1994) observed the attack behavior of this species to collectors of piassava in Amazonas, municipality of Barcelos, suggesting that it was a possible vector of the etiologic agent of Chagas disease, *Trypanosoma cruzi*, which was subsequently confirmed through the finding of human cases (Coura et al. 1995; Junqueira 2005). This species is popularly known as “lice piassava” in the regions Amazonas, Maranhão, and Pará (Rebelo et al. 1998), and its geographic distribution extends along with the palm from Brazilian territory to Venezuela (Lent and Wygodzinsky 1979) and Colombia (D'Alessandro et al. 1971).

Lent and Wygodzinsky (1979) described the morphological and color characteristics of the pronotum of *R*. *brethesi* as having a black posterior lobe between the clear submedian carinae, and clear between these areas and the lateral edges.

**Figure 1.**
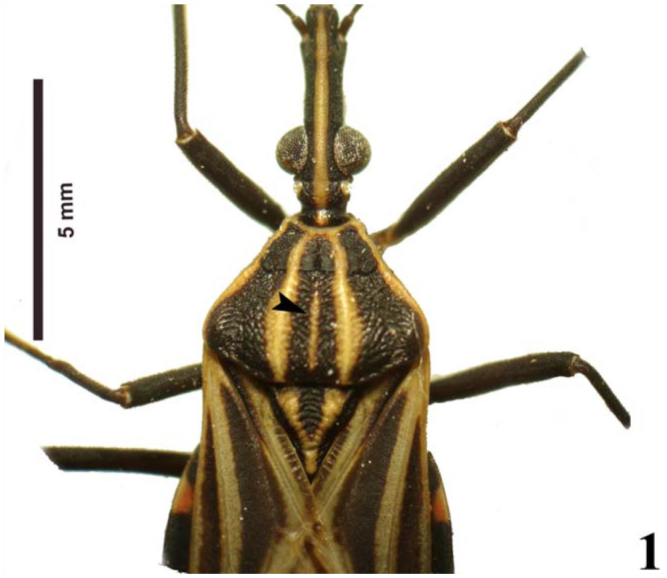
Dorsum of a male *Rhodnius brethesi*, showing in detail the wide medial pronotal band (arrowhead). Photo by Catarina M. Lopes. High quality figures are available online.

**Figure 2.**
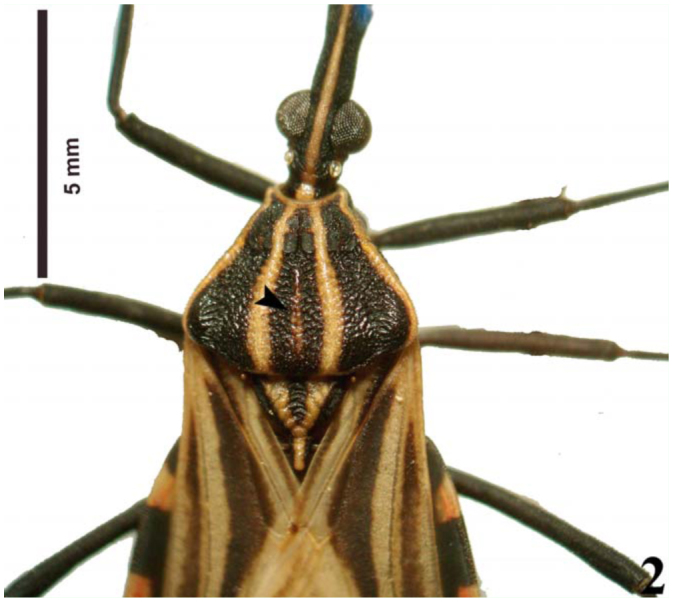
Photo of *Rhodnius brethesi*, showing in detail the narrow medial pronotal band (arrowhead). Photo by Catarina M. Lopes. High quality figures are available online.

**Figure 3.**
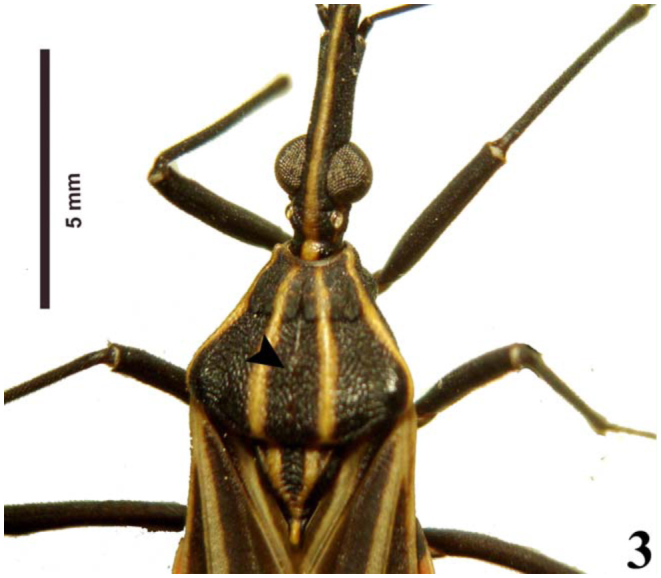
Photo of *Rhodnius brethesi*, showing in detail the absent medial pronotal band (arrowhead). Photo by Catarina M. Lopes. High quality figures are available online.

However, specimens recently collected in the field showed variation of the pattern of the medial pronotal band with three possible states: a wide band (Figure 1), a narrow band (Figure 2), and absent (Figure 3) between the submedian carinae. This observation makes the identification of specimens using the dichotomous key of Lent and Wygodzinsky (1979) difficult, since the variations in the medial pronotal band are not mentioned by the authors.

Since the morphology of testis follicles is an important character in species of Triatominae and can distinguish among species of the same genus, the present study aims to investigate differences in the testis follicles between the two medial pronotal band forms of *R*. *brethesi* (the wide medial pronotal band and the narrow medial pronotal band) testing the following hypotheses: does bilateral symmetry exist between testes and the division of the seven follicles into two groups (short and long) according to general pattern of the *Rhodnius* genus? The results may help determine whether population variations of *R*. *brethesi* exist in the Brazilian Amazon.

## Materials and Methods

*Rhodnius brethesi* specimens were captured from *L*. *piassaba* palm trees located in the tributaries of the left margin of Rio Negro, Barcelos Municipality, Amazonas, Brazil, and kept in the insectary of the Laboratório de Doenças Parasitárias, lnstituto Oswaldo Cruz, FIOCRUZ. Fifth instar male nymphs, sexed according to Lent and Jurberg (1969), were maintained at 29 ± 1^°^C, 80 ± 5% RH and 12:12 L:D in the insectary of the Setor de Entomologia Médica e Forense, lnstituto Oswaldo Cruz, FIOCRUZ, and fed weekly with blood from Swiss mice (Protocolo CEUA - FIOCRUZ P0100-01).

The insects were observed daily and separated according to characteristics of the medial pronotal band, with 30 males exhibiting a wide pattern, and 30 males exhibiting a narrow pattern. The third pattern (absent) was not studied because it is seldom seen.

After the imaginai molt, the newly hatched adults were starved for three days to avoid nutritional effects on the testis development.

The male reproductive tract was dissected in a Petri dish containing saline solution for insects (0.1 M NaCl + 0.1 M KCl). The testes were isolated, identified as left and right testis, and placed in glass lamina filled with the same saline solution. Subsequently, the testis follicles were distended by the disruption of the scrotal membrane and fixed to the lamina using a gentle compression side.

Drawings were made using a steromicroscope with a camera lucida, and measurements were carried out with aid of curvimeter. As some testis follicles were folded at a 90^°^ angle, measurements of the lengths were taken in both sides of each follicle, but only the largest values were considered (Gonçalves et al. 1987). Morphometric data were analyzed using ANOVA at a significance level of 5% using the software R (R 2004).

## Results

**Figure 4.**
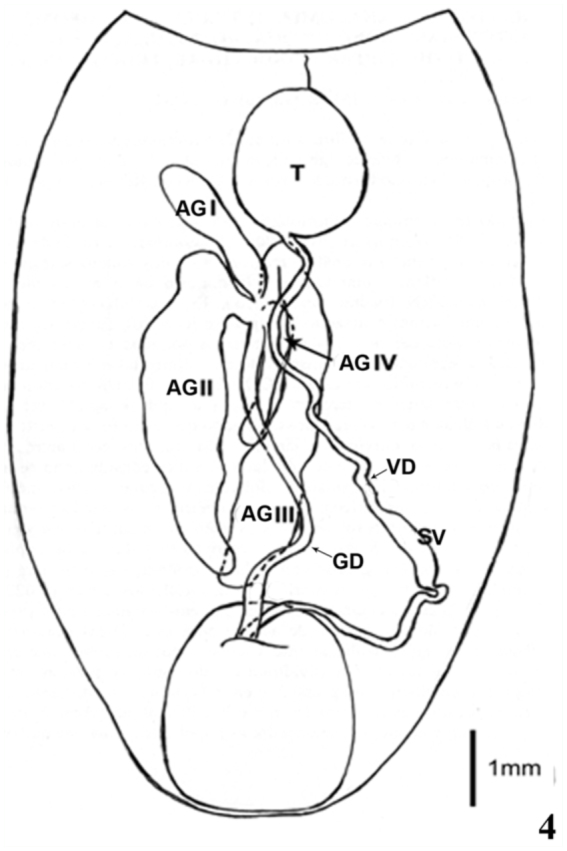
Schematic drawing of the male reproductive tract of *Rhodnius brethesi*. Glandular Duct (GD); Acessory Glands (AG): anterior (AG I), external (AG II), internal (AG III) and dorsal (AG IV); Testes (T); Vas Deferens (VD); Seminal Vesicle (SV). High quality figures are available online.

The male reproductive tract of *R*. *brethesi* is comprised of a pair of testicles, two vas deferens, two seminal vesicles, and one ejaculatory duct that terminates in the aedeagus. Four pairs of accessory glands (anterior, external, internal, and dorsal glands) join at a point forming the glandular duct ending in the vas deferens. With regard to the morphology of the accessory glands, glands I (anterior) and IV (dorsal) are smaller compared to glands II (external) and III (internal), which are noticeably larger (Figure 4).

**Table 1.**
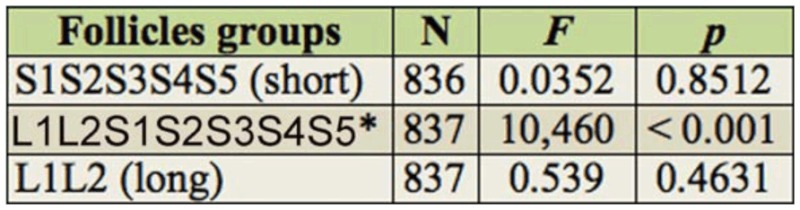
Analysis of variance (ANOVA) of the testis follicles in the *Rhodnius brethesi*.

**Figure 5.**
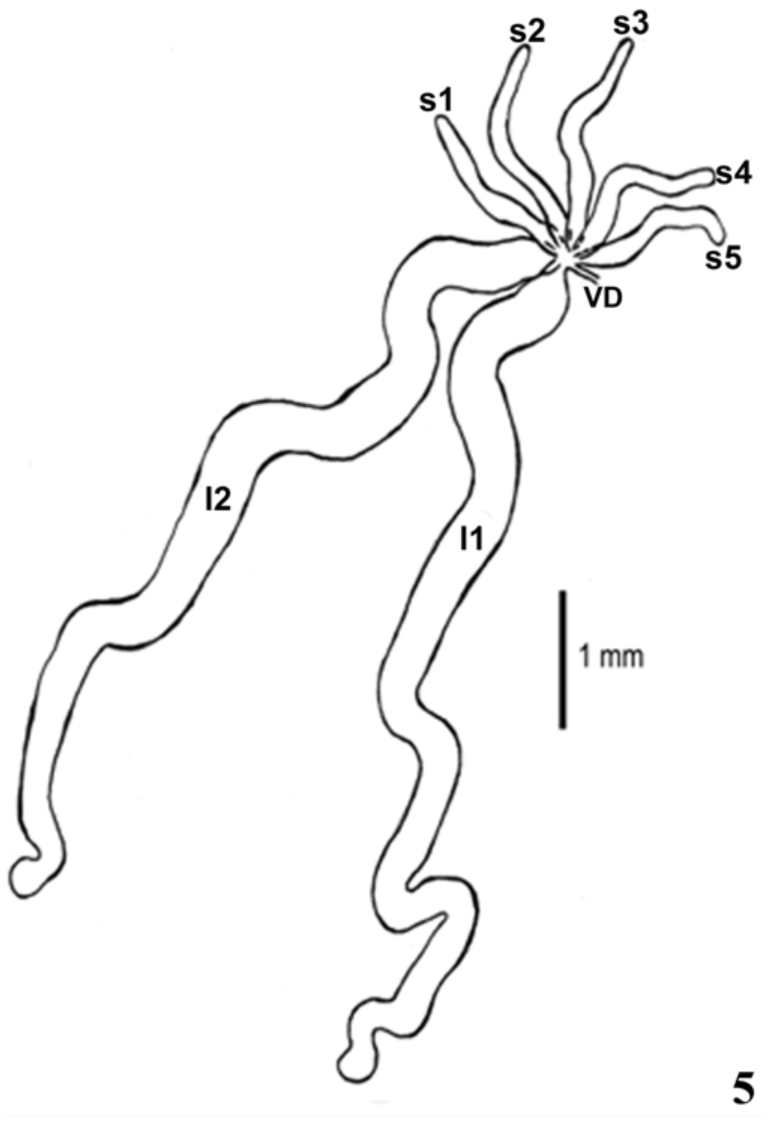
Schematic drawing of the testicular follicles of *Rhodnius brethesi*. Long follicles (II ans 12); short follicles (s1–s5); Vas Deferens (VD). High quality figures are available online.

**Figure 6.**
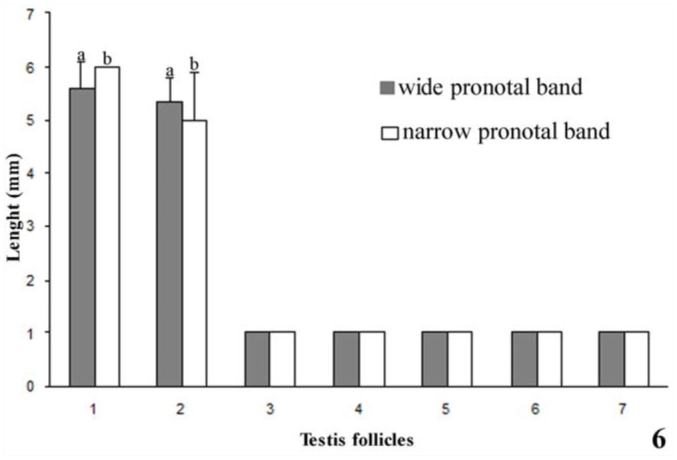
Length (mm ± SD) of the seven testis follicles in *Rhodnius brethesi*. Different letters in the bars indicates significant differences by the F test at a 5% significance level. High quality figures are available online.

*Rhodnius brethesi* has seven testis follicles, two long (11 and 12) and five short (s1–s5) (Figure 5). The follicles are variable in length (1.1–6.45 mm) and are similar between left and right testes (ANOVA, *F*_(831)_ = 0.7217; *p*= 0.40). The statistical analysis allowed the classification of the follicles into two distinct groups, one with two long follicles (11 and 12) (ANOVA, *F*_(837)_= 0.54; *p*= 0.4631) and the other with five short follicles (s1–s5) (ANOVA, *F*_(836)_= 0.035; *p*= 0.85) (Figure 6, Table 1).

**Figure 7.**
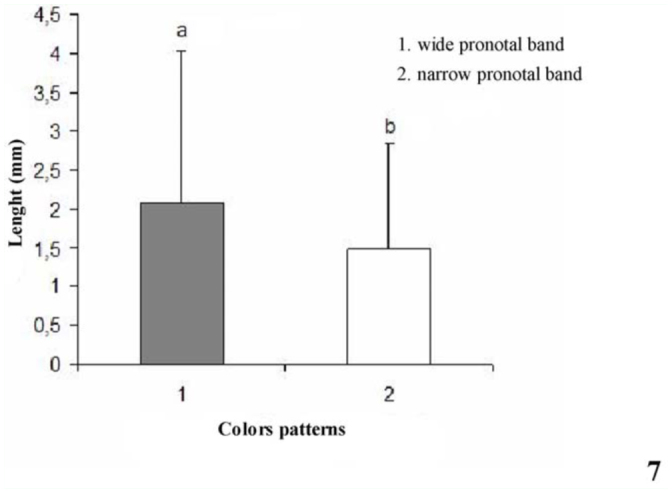
Length (mm) of the two groups of testis follicles in *Rhodnius brethesi*. Different letters in the bars indicates significant differences by the F test at a 5% significance level. High quality figures are available online.

Statistical analysis showed that follicles with a wide medial pronotal band were grouped into long (> 5.4 mm) and short (1 mm) morphology, and follicles with a narrow medial pronotal band measured long (5.64 mm) and short (1 mm), demonstrating that there is significant difference between them (ANOVA,*p* < 0.01) (Figures 6 and 7).

## Discussion

Because of the diversity of biological and ecological patterns, the insects have anatomical differences in the structures associated with reproduction (Chapman 1998; Triplehorn and Jonnson 2011). Thus, interspecific variation in reproductive systems may occur in the size or number of structures, in absence of any of them, or in their position along the reproductive tract (Adiyodi and Adiyodi 1975; Grassé 1982; Chapman 1998).

The general aspect of the male reproductive tract of *R*. *brethesi* follows as described for other Triatominae (Barth 1958; Freitas et al. 2008). In *Triatoma* it is possible to note that all four male accessory glands show variation between them; glands I and II are larger compared to glands III and IV, which are noticeably smaller (Freitas et al. 2008, 2010). In this study, glands II and III are larger compared to the glands I and IV (noticeably smaller), suggesting the existence of interspecific variation.

The substances of the male accessory glands among insects in general are related with sperm protection, storage and activation, sperm competition, female behavior (reduction in attractiveness), fecundity, ovulation, oviposition, and protection of laid eggs (Davey 1958; Fuchs et al. 1969; Pickford et al. 1969; Hartmann and Loher 1999; Gillot 2003; South et al. 2008). In *Rhodnius prolixus*, the spermatophore is produced by the three pairs of male accessory glands that contain a transparent material, while the secretion of the fourth pair, of opaque aspect, is responsible for the movement of the spermatozoa inside of the reproductive organ of the female (Davey 1958). In a recent study, Freitas et al. (2011) showed that in *T*. *brasiliensis*, the AG III and AG IV do not participate in the production of carbohydrates until the fifth day of adult life, suggesting that one or both of these glands may play the same role of the opaque glands of *R*. *prolixus*. Considering this, it can be inferred that interspecific morphological variation of the accessory glands in Triatominae may be related to their secretory products, as noted in *Luciola* fireflies, which have three morphological types of accessory glands, with only the curled glands being responsible for producing spermatophore precursors (South et al. 2008).

Studies of reproductive behavior in Heteroptera are few. Mating systems have been described among Gerromorphans: monopolization of the females through postcopulatory guarding (VepsäHänen 1985; Murray and Giller 1990) or resource defense polygyny through male territoriality and defense of oviposition (Hayashi 1985; Nummelin 1988). Since male reproductive success varies directly with the number of matings, is possible that a smaller number of follicles can lead to a low rate of sperm production, providing defenses against male polygyny, as post—copulatory guarding, observed in species of the Hebridae, Hydrometridae, Mesoveliidae, and Veliidae that has only one follicle, and Gerridae with two follicles (Woodward 1950; Pendergrast 1956). Thus, a smaller number of follicles can lead to a low rate of sperm production, providing defenses against male polygyny, as post—copulatory guarding. In contrast, males of Geocorisae (Cimicomorpha + Pentatomorpha) have seven follicles (Woodward 1950; Pendergrast 1956; Akingbohungde 1983; Gonçalves et al. 1987; Lemos et al. 2005; Freitas et al. 2008), which is considered a plesiomorphic condition for this group. Males of some species in this family have shown postcopulatory guarding only in the presence of other males, preventing subsequent copulations of the female (Carroll 1991; Schölf and Taborsky 2002; Vitta and Lorenzo 2009). In these cases, a higher number of follicles can lead to a higher rate of sperm production, maintaining the reproductive success of males and presenting behavior changes only when necessary.

Within Reduviidae, the Triatominae have different lengths and widths of the seven testis follicles in the genera *Panstrongylus*, *Rhodnius*, *Psammolestes*, *Triatoma*, and *Mepraia* (Schreiber et al. 1968; Silva and Schreiber 1971; Gonçalves et al. 1987; Lent et al. 1994). In this study, the length of the follicles of *Rhodnius* confirms the pattern established in previous studies as long and short.

No significant differences in follicle lengths between the left and right testes were observed, supporting the hypothesis of bilateral symmetry see in other species, as well as their classification in two categories: two long and five short from *Rhodnius*. These results confirm those found for *R*. *ecuadorensis*, *R*. *nasutus*, *R*. *neglectus*, and *R*. *prolixus* (Gonçalves et al. 1987).

Freitas et al. (2007) found that *T*. *rubrofasciata* have four categories of follicles: one long, two medium, two short, and two very short, differing from the other species of this genus studied. According to the authors, this character is based on the fact that *T*. *rubrofasciata* is the sister group of *Linshcosteus*, both sister taxa of the remaining Triatomini (Hypsa et al. 2002).

Morphometric analysis of testis follicles in different color patterns of *T*. *brasiliensis* was performed to verify the presence of different size categories of follicles among species of the genus (Freitas et al. 2008). The results showed that the four forms of the brasiliensis complex were separated in three groups: brasiliensis, melanica, and juazeiro + macromelasoma, corroborating previous studies that showed morphological variation in the egg exochorion, isoenzimatic characterization, and genetic distance (Costa et al. 1997a, 1997b; Costa et al. 2003), where these forms have been classified as three species *T*. *brasiliensis*, *T*. *melanica*, and *T*. *juazeirensis*, and one subspecies *T*. *brasiliensis macromelasoma* (Costa et al. 2003, 2006; Costa and Félix 2007). These data emphasize the importance of morphometric studies as an additional character for taxonomic analysis in Triatominae.

Medial pronotal band variations were observed in *R*. *brethesi*. The confirmation of significant differences between the two patterns suggests the existence of populations and possibly a “brethesi” complex, since there are external and internal morphological variations between both patterns.

Studies with morphometric, molecular, and biochemical techniques are underway to better understand these different medial pronotal band patterns in *R*. *brethesi*. The results of these multidisciplinary studies can help to emphasize the need for a revision of the dichotomous key of Lent and Wygodzinsky (1979), since the medial pronotal band patterns may facilitate species identification.
